# Case Report: Simultaneous Hyperprogression and Fulminant Myocarditis in a Patient With Advanced Melanoma Following Treatment With Immune Checkpoint Inhibitor Therapy

**DOI:** 10.3389/fimmu.2020.561083

**Published:** 2021-02-02

**Authors:** Whitney Barham, Ruifeng Guo, Sean S. Park, Joerg Herrmann, Haidong Dong, Yiyi Yan

**Affiliations:** ^1^ Department of Immunology, Mayo Clinic, Rochester, MN, United States; ^2^ Medical Scientist Training Program, Mayo Clinic, Rochester, MN, United States; ^3^ Dermatopathology Section, Division of Anatomic Pathology, Department of Laboratory Medicine and Pathology, Mayo Clinic, Rochester, MN, United States; ^4^ Department of Radiation Oncology, Mayo Clinic, Rochester, MN, United States; ^5^ Department of Cardiovascular Diseases, Mayo Clinic, Rochester, MN, United States; ^6^ Department of Urology, Mayo Clinic, Rochester, MN, United States; ^7^ Division of Medical Oncology, Mayo Clinic, Rochester, MN, United States

**Keywords:** melanoma, immune checkpoint inhibitors, hyperprogression, myocarditis, cytolytic T lymphocytes, case report

## Abstract

We report here a patient with stage IV mucosal melanoma treated with dual immune checkpoint inhibitor (ICI) therapy (Nivolumab/Ipilimumab) who experienced rapid disease progression and metastatic spread within three weeks of first infusion. Surprisingly, this patient also developed fulminant myocarditis within the same time frame. Immunohistochemical staining of the primary tumor and a metastatic omental lesion revealed robust CD8^+^ PD-1^+^ T cell infiltration after ICI treatment, as would be expected following immune activation. However, the CD8^+^ T cell infiltrate was largely negative for both Granzyme B and TIA-1, suggesting these T cells were not capable of effective tumor lysis. We discuss the possibility that heightened pro-inflammatory T cell activity (rather than tumor-directed cytolytic activity) was induced by anti-PD-1 and anti-CTLA-4, which could have provoked both rapid tumor resistance mechanisms and myocarditis. This case highlights the fact that the mere presence of tumor infiltrating lymphocytes (TILs) does not necessarily correlate to ICI response and that additional functional markers are necessary to differentiate between inflammatory and cytolytic CD8^+^ TILs.

## Introduction

Cytotoxic T lymphocyte-associated antigen 4 (CTLA-4), programmed cell death protein 1 (PD-1), and programmed cell death 1 ligand 1 (PD-L1) are immune-regulatory molecules which function to limit adaptive immune activity within the normal host. However, these “checks” on the immune system can be coopted by malignant cells to create an immunosuppressive environment and evade apoptosis induced by cytolytic T cells. Monoclonal antibodies that bind to CTLA-4, PD-1, and PD-L1 are designed to block these proteins from interacting with their respective binding partners, and are collectively known as immune checkpoint inhibitors (ICIs). Since their approval in 2011 (anti-CTLA-4) and 2014 (anti-PD-1/L1), ICIs have revolutionized the treatment of solid tumor malignancies including melanoma, non-small cell lung cancer, renal cell carcinoma, and gastric cancer, among others (1–6). A number of trials have reported striking results, as patients with stage IV metastatic cancers have had significant responses and achieved remission, some for extended periods of time (7, 8). However, the majority of patients treated do not respond, and some suffer significant adverse events such as inflammation of normal, non-tumor tissue (9). In addition, patients may experience *accelerated* growth of their tumor following ICI therapy, a phenomenon that has been termed hyperprogression (9–11). At present we cannot predict which patients may benefit from ICI and which patients will develop adverse effects.

The case presented here uniquely highlights this gap in knowledge, as our patient experienced both rapidly progressive metastatic disease and a life-threatening immune related adverse event (myocarditis) within three weeks of initiating ICI therapy. Our current understanding would predict that induction of a systemic immune response strong enough to manifest as myocarditis should lead to a clinical response in the form of tumor shrinkage. In this case, the opposite occurred, as evidenced by aggressive tumor spread. This emphasizes the urgent need for a better understanding of what factors are critical for coupling the immune response induced by ICI to a positive clinical outcome. We propose that the phenotype of CD8^+^ T cells activated by ICI therapy may be key in this determination.

### Case Presentation

**Figure 1:**
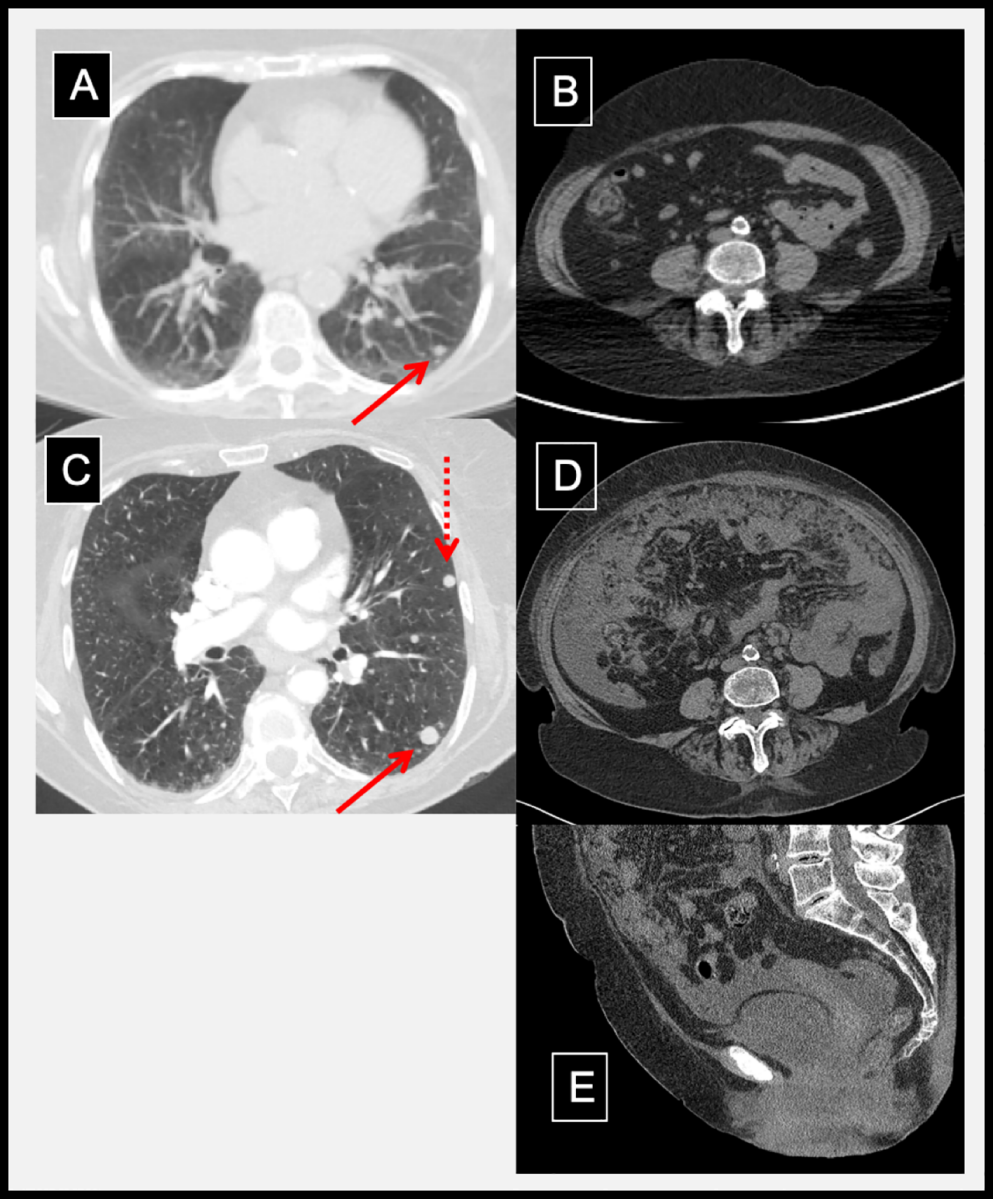
Radiographic evidence of hyperprogression and inflammation following dual immune checkpoint inhibition. Pre-treatment CT chest with a lung metastasis **(A)**; solid arrow) and a normal abdominal CT **(B)**. Three weeks after ipilimumab and nivolumab, prior lung metastasis grew (**C**; solid arrow) and new metastasis developed (**C**; dotted arrow). In addition, abdominal distention, omental caking, peritoneal metastases, and pelvic ascites developed as shown in axial **(D)** and mid-sagittal **(E)** abdominal/pelvic CT.

In October 2018, a 79-year-old female experienced new onset postprandial abdominal cramping. Over the next several days, she noted constipation, dark stools, and vaginal bleeding. This prompted her to visit her local primary care provider who detected a vaginal wall lesion during physical exam, and excisional biopsy confirmed malignant mucosal melanoma. Staging PET/CT and pelvic MRI confirmed a 2.5 × 2.7 × 2.9 cm mass in the vaginal mucosa and pre-sacral lymph nodes which were later biopsy-confirmed metastases. No other distant metastasis was found. After this initial diagnosis and workup elsewhere, she presented to our clinic in February 2019 for a second opinion having received no prior treatment. The patient’s medical history, review of systems and physical exam at the time were unremarkable, except for moderate vaginal bleeding and discomfort near coccyx. Her ECOG performance was 0. Due to > 2 months lapse since her diagnosis without initiation of therapy, she was restaged with PET/CT which demonstrated distant osseous and visceral metastases involving lungs as well as progression of distant and regional nodal metastases and primary vagina melanoma (**Figures 1A, B**). Lactate dehydrogenase (LDH) level was elevated (564 U/L, upper limit of normal is 222 U/L). Foundation One^®^ testing was performed on the primary tumor specimen, which revealed CDKN2A deletion, with no other mutations or alterations.

A single fraction palliative radiotherapy was delivered to vagina (10 Gy) and pelvic/para-aortic lymph nodes (8 Gy) to alleviate her vaginal bleeding and pelvic discomfort, respectively. Systemic therapy with ICI (3 mg/kg Nivolumab + 1 mg/kg Ipilimumab) was initiated concurrently. First infusion of ICI was uneventful, and patient returned three weeks later for follow-up and subsequent ICI cycle. LDH level remained elevated (365 U/L). Two days after her second infusion, she presented to the emergency department (ED) with complaints of dizziness, a light-headed “spell,” and abdominal bloating. She was hypoxic, and chest, abdominal, and pelvic CT showed rapid progression of known and new metastatic disease including omental caking, peritoneal metastases, pelvic ascites, and lung nodules (**Figures 1C–E**). CT-guided core biopsy of an omental mass confirmed metastatic melanoma. Given her rapid disease progression, carboplatin (AUC 2, day 1) and paclitaxel (60 mg/m^2^, days 1, 8, 15 every 28 days) was initiated as salvage therapy after the patient was medically stabilized.

Two days later, the patient presented again to the ED following a syncopal episode. While in the ED, she had two episodes of bradycardia in the 20s and progressed to complete heart block. She was given IV atropine and transferred to the Cardiac Critical Care Unit for an emergent pacemaker placement. Poor R wave sensing and consistently high thresholds upon multiple lead repositioning attempts caused the team to consider myocarditis, likely induced by ICI, which was later confirmed *via* endomyocardial biopsy. The patient was stabilized, but remained critically ill. Based on goals of care, she was discharged on corticosteroids to hospice care and passed away shortly afterwards.

**Figure 2:**
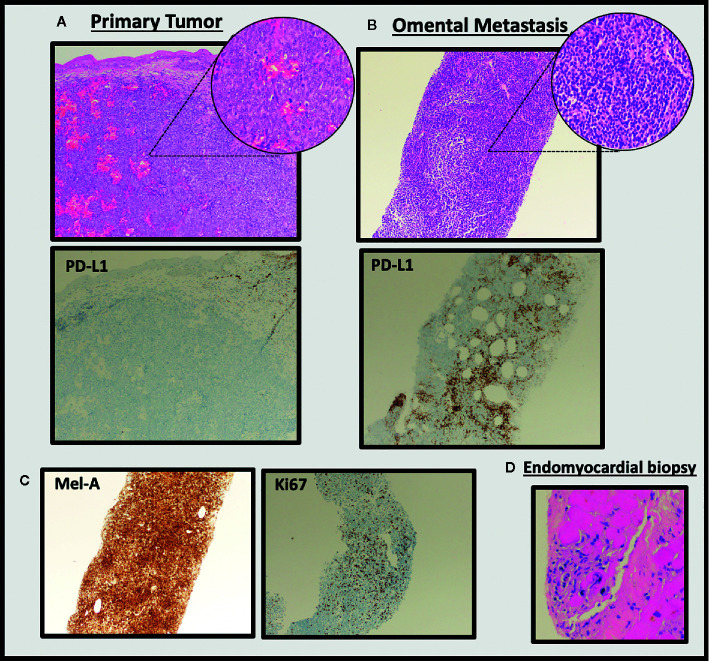
Histological evaluation of tumor tissue and endomyocardial biopsy. H&E staining of the pre-treatment primary vaginal melanoma specimen **(A)** reveals epithelioid morphology and largely negative staining for PD-L1. This is in stark contrast to the metastatic omental specimen from three weeks post-ICI **(B),** which exhibited small cell morphology and was positive for PD-L1 (100× images, insets are 400×; clone 22C3 used for PD-L1 stain). The omental metastasis stained positive for Mel-A and Ki67 (**C,** 100× images). Endomyocardial biopsy confirms myocarditis (**D**, 100×).

Considering the significant disease progression within just three weeks of initiating dual ICI, we pursued further investigation of the tumor biopsy samples. Analysis of the initial primary vaginal melanoma specimen revealed epithelioid morphology, and tumor cell staining for PD-L1 was almost completely negative (**Figure 2A**). In contrast, the metastatic omental specimen from three weeks post-ICI exhibited small cell morphology, mimicking lymphocytes, and approximately 10% of tumor cells within the metastasis stained positive for PD-L1. Due to the drastic difference in morphology, we completed an additional stain demonstrating strong positivity of the metastatic tumor cells to a specific melanocytic marker (Mel-A), which confirmed the biopsy tissue was indeed metastatic melanoma (**Figures 2B, C**). Also consistent with rapid clinical and radiographic disease progression, the omental lesion was highly proliferative with 60% of cells staining positive for Ki67 (**Figure 2C**). In addition to progressive tumor expansion at metastatic sites, this patient also experienced the onset of grade 4 myocarditis which was confirmed by endomyocardial biopsy of the right ventricle. The biopsy demonstrated a solitary focus of interstitial lymphocytic infiltrate in association with focal myocyte injury (**Figure 2D**).

**Figure 3:**
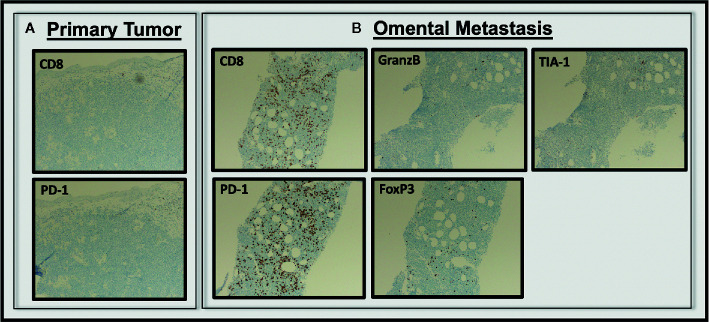
Analysis of T cell infiltrate in pre and post treatment tumor tissue. CD8^+^, PD-1^+^ cells were essentially absent from the pre-treatment primary vaginal melanoma specimen **(A)**, but were abundant in the metastatic omental specimen from three weeks post-ICI **(B)**. The CD^+^PD-1^+^ T cells within the omental metastasis were largely negative for Granzyme B, T1A-1, and Foxp3 (All images 100×).

Assessment of the T cell infiltrate within the tumors revealed minimal CD8^+^ or PD-1^+^ T cells in the primary vaginal lesion (5–10% and 1% of total cells, respectively) (**Figure 3A**), which were predominantly distributed in the areas adjacent to rather than within the tumor foci. Interestingly, the metastatic omental specimen contained a much more robust CD8^+^ T cell infiltrate (~30% of total cells), which also showed a consistent staining pattern with PD-1, suggesting that the T cells were antigen-engaged (12, 13) (**Figure 3B**). Importantly, these lymphocytes were completely distributed within tumor foci. However, only a minority of the CD8^+^ T cells stained positive for the cytolytic granule protein Granzyme B (a serine-protease that mediates apoptosis in target cells) or TIA-1 (an RNA-binding protein associated with cytolytic granules), suggesting the CD8^+^ T cells were not capable of tumor cell killing. The presence of FoxP3^+^ cells was also assessed as others have reported that immunosuppressive regulatory T cells (Tregs) could be responsible for rapid progression following ICI (14). Treg component was minimal at approximately 3% of total cells in the most densely staining area (**Figure 3B**).

## Discussion

**Figure 4:**
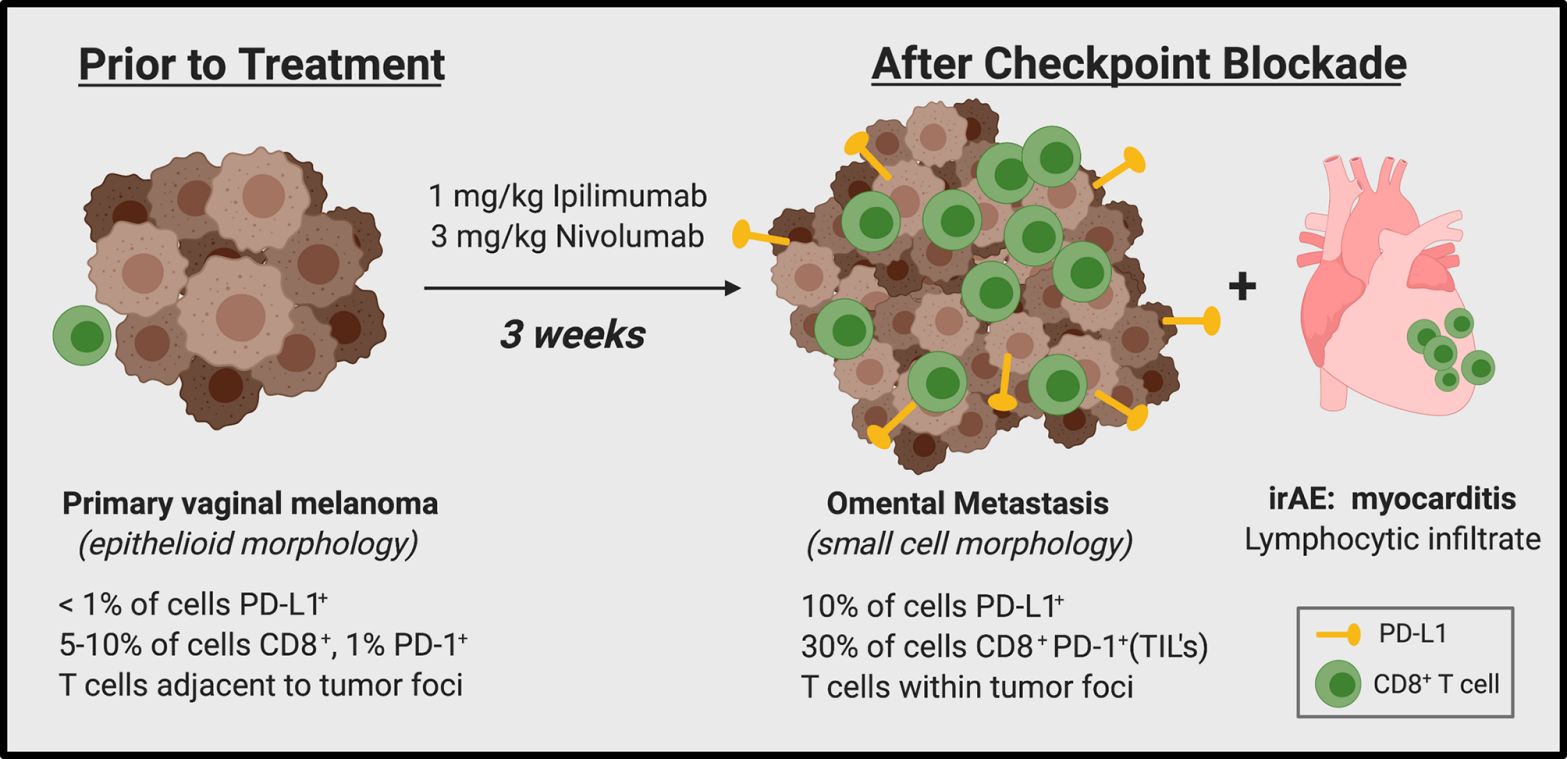
Summary of case.

Herein we present a case of metastatic mucosal melanoma with rapid disease progression and myocarditis within three weeks of initiation of dual ICI therapy (**Figure 4**). Such phenomenon of accelerated tumor growth within months of starting ICI has recently been recognized and referred to as hyperprogression (9–11). The definition of hyperprogression is not widely agreed upon. Some use measurements of a target lesion over multiple time points to compute the pretreatment growth rate of the tumor vs. growth rate while on therapy, but the series of scans spaced at appropriate timepoints necessary to calculate these metrics limits the pool of patients that can practically be assessed. Lo Russo et al. used a more inclusive definition which categorizes patients as having hyperprogressive disease if they meet three of five clinical criteria (15). Our patient met three of these benchmarks, including: (i) time-to-treatment failure < 2 months; (ii) spread of the disease to a new organ between baseline and first radiologic evaluation; (iii) and clinical deterioration with decrease in ECOG performance status ≥ 2 during the first 2 months of treatment.

No common tumor mutations or disease characteristics have been identified among those who experience any form of this early, aggressive tumor growth while on ICI, and little has been elucidated in terms of a mechanism for how this may happen. In fact, the occurrence of true ICI-induced hyperprogression has been questioned altogether, as the accelerated tumor growth could be the result of factors intrinsic to the tumor itself which would lead to rapid growth over time even without the initiation of ICI (16). To this end, recent communication from Bristol Meyer Squibb highlights data from their CheckMate 451 and ATTRACTION-2 clinical trials which show that hyperprogression can be found at similar incidence in both placebo and ICI-treated patients (17, 18).

Though there is controversy surrounding whether ICI-directly causes rapid tumor progression in any or all of the “hyperprogression” cases in the literature, there is still relative agreement on the fact that a small subset of patients do very poorly on ICI, and progress quickly, whatever the root cause. Thus, even if ICI is not having a direct detrimental effect, it is obvious that some tumors are able to continue growing rapidly, un-checked by the immune system, even though therapy is being given to stimulate an immune response. Some might argue that in these cases no immune response is ever being induced, perhaps due to lack of appropriate antigen expression or failure of lymphocytes to infiltrate into the tumor. That is why we found this case particularly interesting: there was an obvious immune related side-effect (myocarditis) which confirms that at least systemically, the ICI therapy had a strong immune-stimulatory impact. Thus, the question remains: why was this immune response so ineffective? It was unable to contain the tumor, despite creating a massive inflammatory side-effect in the cardiac tissue. Below we discuss possible immunological mechanisms that would connect an immune response induced by ICI to the development of both the aggressive metastatic disease as well as the myocarditis. While there are some supportive lines of evidence for this from the literature, it should be stated that we certainly cannot prove a causal connection.

It has been reported that anti-PD-1 therapy could be paradoxically enhancing immunosuppression through Tregs or myeloid cells, rather than enhancing anti-tumor activity through CD8^+^ T cells (14, 15). This is in contrast to our patient, as analysis of post-treatment tumor tissue revealed an active immune environment with scant Tregs and significantly greater numbers of CD8^+^ TILs. These TILs appear to have been stimulated with antigen, given their PD-1^+^ status, and were likely secreting cytokines, given the up-regulation of PD-L1 on the tumor cells, which can be seen in response to IFN-gamma secretion by CD8^+^ T cells (19, 20). Though CD8^+^ T cells are thought to be the primary mediators of the anti-tumor effects following ICI, it is apparent that a mere increase in tumor infiltrating cell counts does not correlate with better prognosis (21). This may be because all CD8^+^ T cells are not equal in their ability to eliminate tumors. Indeed, recent studies have suggested that an entire spectrum of PD-1^+^, antigen-stimulated CD8^+^ T cells exist, with phenotypes that range from “stem-like” to “effector” to “truly exhausted,” all of which differ in their cytolytic abilities (22, 23). Thus, it is plausible that certain patients possess a group of T cells that are both tumor-specific and PD-1 positive, but functionally unable to kill. In the presence of anti-PD-1 or anti-CTLA-4 therapy these cells may increase their cytokine production, kindling the tumor rather than killing it. Stein et al. recently tested this hypothesis in a breast cancer model, in which they show that tumor cell interactions with nonlytic T cells, which secrete inflammatory cytokines but lack cytolytic granules, were able to induce signatures characteristic for adaptive immune resistance and tissue regeneration in breast cancer cells (24). These changes were induced rapidly (within hours of co-culture), and resulted in faster tumor outgrowth and increased metastatic spread when the cells were injected *in vivo*. Thus, if inflammatory signaling outweighs cytolytic competency, CD8^+^ T cells themselves could lead to tumor resistance and accelerated tumor progression. Interestingly, our staining showed an absence of both Granzyme B and TIA-1 staining within the CD8^+^ T cells infiltrating the metastatic lesion, suggesting they lack tumor-killing capabilities and could have instead been the source of cytokines that induced rapid resistance.

Kim et al. provide additional evidence that the relative abundance of different functional phenotypes of T cells prior to therapy can determine whether a tumor might hyperprogress. Using 144 pre-treatment peripheral blood samples from patients with NSCLC, they found that lower frequency of effector/memory subsets (CD8^+^CCR7^−^CD45RA^−^) and higher frequency of “severely exhausted” (CD8^+^TIGIT^+^PD-1^+^) T cells were associated with hyperprogressive disease (25). Similarly, changes in CD4^+^ T cell profiles before and after treatment differentiated responders, non-responders, and hyperprogressors in a prospective study of NSCLC patients (26). Thus, the findings from our case as well as these larger cohorts highlight the need for better immune-monitoring tools that can assess the functional capabilities of T lymphocytes in tumor biopsies and in the peripheral blood prior to initiation of ICI. Here we have used Granzyme B and TIA-1, which have been used previously to assess cytolytic capacity, but additional tools are needed (27, 28). Ideally, we would have the ability to determine not only the number and frequency of CD8^+^ T cell subsets, but also whether they contain the appropriate machinery to induce cytotoxicity once activated by ICI. This could be important for identifying those patients who may hyperprogress and also for determining what molecules need to be restored in T cells in order to increase positive response rates to these therapies.

While experiencing rapid tumor growth and metastatic spread, our patient also developed fulminant myocarditis secondary to immune checkpoint blockade. Several studies have shown a correlation between immune-related adverse events (irAEs) and response to ICI, while others have found no such relationship (29, 30). Myocarditis is an irAE observed in approximately 1% of patients on ICI therapy (31, 32). It most often occurs in the first 30 days after initiation of therapy, as it did in our patient (32). To our knowledge this is the first report of simultaneous rapid acceleration of tumor growth and irAE myocarditis experienced by the same patient. Given the immune status of the metastatic lesion, this may reflect heightened inflammatory T cell activity, induced by anti-PD-1 and anti-CTLA-4, which could have resulted in both rapid tumor resistance mechanisms and myocarditis. The number, type, and timing of irAEs experienced by patients who hyperprogress have not been reported in the studies published to date. It would be interesting to determine if a subset of hyperprogressors also experience rapidly induced irAEs, and if these patients perhaps share a common hyper-inflammatory mechanism.

Though we were able to compare staining of the pre-treatment and post-treatment samples from this patient for a number of immune-relevant markers, tissue sections were limited, especially of the primary tumor and the endomyocardial biopsy. This prevented us from doing further analysis of myeloid populations or cytokine molecules and from assessing clonality of the T cells infiltrating the tumor tissues vs. the heart to determine if they were related. We also lacked pre- and post-treatment peripheral blood samples which would have facilitated comparison of immune cell populations *via* flow cytometry with the immunohistochemistry (IHC) staining in the tumor tissues. Despite these limitations, this case provides intriguing evidence that suggests CD8^+^ T cell infiltration and activation can be concurrent with rapid disease progression following ICI. We conclude that assessment of inflammatory vs. cytolytic function of CD8^+^ T cells may be critical for determining why some tumors continue to progress despite ICI-therapy, and could give insight into why patients experience severe irAEs.

## Data Availability Statement

The raw data supporting the conclusions of this article will be made available by the authors, without undue reservation.

## Ethics Statement

The studies involving human participants were reviewed and approved by Mayo Clinic Institutional Review Board. The patients/participants provided their written informed consent to participate in this study. Written informed consent was obtained from the individual(s) for the publication of any potentially identifiable images or data included in this article.

## Author Contributions

YY was the patient’s medical oncology provider and initiated the research. SP completed the radiological analysis. RG completed the histological and pathological tissue analysis. WB wrote the paper. WB, RG, SP, JH, HD, and YY critically evaluated data, reviewed the paper, and approved the final version of the article to be published. All authors contributed to the article and approved the submitted version.

## Funding

This work was supported by K12CA090628 (YY), The Richard M. Schulze Family Foundation Award in Cancer Research (HD), R01 CA 200551 (SP and HD), P30 CA 15083 (HD).

## Conflict of Interest

The authors declare that the research was conducted in the absence of any commercial or financial relationships that could be construed as a potential conflict of interest.
